# Characterization of the *Pank2*^-/-^ mouse retinal phenotype as a pre-clinical model for pantothenate kinase-associated neurodegeneration

**DOI:** 10.1371/journal.pone.0326866

**Published:** 2025-06-24

**Authors:** Grace Li-Na Su, Suh Young Jeong, Dahlia Wafai, Wayne Tschetter, Dolly Zhen, Susan J. Hayflick, Renee C. Ryals

**Affiliations:** 1 Department of Ophthalmology, Casey Eye Institute, Oregon Health and Science University, Portland, Oregon, United States of America; 2 Department of Molecular and Medical Genetics, Oregon Health and Science University, Portland, Oregon, United States of America; Eye Foundation Hospital / Eye Foundation Retina Institute, NIGERIA

## Abstract

Pantothenate kinase-associated neurodegeneration (PKAN) is an autosomal recessive movement and vision disorder in the neurodegeneration with brain iron accumulation family of diseases. PKAN is caused by mutations in *PANK2*, encoding pantothenate kinase 2, causing an inborn error of coenzyme A metabolism and leading to iron accumulation in the basal ganglia. Peripheral pigmentary retinopathy is common in people with PKAN. The knockout murine model of the orthologous *Pank2* gene is known to manifest retinal degeneration through electroretinography, pupillary response and histology analyses. Our longitudinal characterization of the retinopathy in this model reveals reduced visual performance and reduced photoreceptor thickness compared to wild-type mice. Additionally, retinal perturbations in coenzyme A metabolism and dopamine metabolism pathways mimic those previously observed in the brain. These data extend the murine ocular phenotype associated with loss of function of Pank2. With a measurable behavioral, structural and mechanistic retinal phenotype, this mouse model is an ideal pre-clinical model that can be used to evaluate therapeutics for PKAN.

## Introduction

Pantothenate kinase-associated neurodegeneration (PKAN) is a rare disorder characterized by dystonia, parkinsonism, retinopathy, brain iron accumulation, and early death in children and adults [1]. PKAN is caused by mutations in the gene encoding pantothenate kinase 2 (PANK2), one of four isozymes and the only one that localizes to mitochondria. Pantothenate kinases, including PANK1ɑ, PANK1β, PANK2 and PANK3, catalyze the phosphorylation of pantothenate and pantetheine in coenzyme A (CoA) synthesis. Although the precise pathogenesis of PKAN remains unclear, evidence suggests that PANK2 serves a critical role specifically in supporting the phosphopantetheinyl-activation of mitochondrial proteins, including mitochondrial acyl carrier protein [2–4].

Many people affected with PKAN manifest significant visual impairment from pigmentary retinopathy. In case studies predating gene discovery, descriptions of retinal pathology of people most likely with PKAN and diagnosed with the now discarded eponym “Hallervorden-Spatz syndrome,” (now referred to as neurodegeneration with brain iron accumulation (NBIA) diseases) include retinal arteriole attenuation, hyperpigmented foveas, peripheral bone corpuscle pigmentation, enlarged retinal pigmented epithelium cells (RPE), loss of photoreceptor outer and inner segments, thinned or absent outer nuclear layer (ONL), macrophage infiltration of the ONL, and melanolipofuscin aggregation under the retina [5,6]. In recent data obtained from patients with PKAN at Oregon Health & Science University, two-thirds of those with classic PKAN, the earlier onset more aggressive childhood form, had impaired night vision and about half had been diagnosed with an eye condition, while 43% of people with atypical PKAN, the later onset more insidious form, reported impaired night vision and only 19% had been diagnosed with an eye condition. In a more recent series looking at neuro-ophthalmic findings in people with PKAN, five out of ten had abnormal vertical optokinetic responses, eight out of ten had Adie’s pupil phenotype in both eyes, and 11 out of 16 patients had abnormal electroretinograms (ERGs), although only four out of ten patients had frank pigmentary retinopathy (retinitis pigmentosa) evident on dilated funduscopic examination [7]. A recent study of the retinal neurovasculature in people with an NBIA disorder found retinal thinning specifically in PKAN, with particularly significant degeneration of the outer nuclear layer (ONL) compared to controls. More than half of those with PKAN had abnormal ERGs, but there was no difference found in retinal vessel densities [8]. These data provide critical insights into the PKAN retinopathy phenotype, though there is more to learn.

As a disease model, *Pank2*^-/-^ mice manifest a robust constellation of abnormalities that align strongly with key features of PKAN in humans. These include defects in CoA, iron and dopamine metabolism specifically in the region of brain correlating with the site of disease in humans, the globus pallidus [2]. These biochemical and functional changes have revealed valuable insights into disease pathophysiology, even though the animals show no clinical neurological features. In addition, these animals were previously shown to manifest retinal degeneration through electroretinography, pupillary response, and histology analyses [9], features that again largely recapitulate the findings in the human retinopathy. Together the brain and ocular data represent tractable candidate outcome measures.

Although management of people with PKAN is still largely symptomatic, potentially impactful therapeutics are advancing. A ‘bypass’ molecule that corrected CoA, iron and dopamine metabolic defects in the brain of *Pank2*^-/-^ mice is in development for humans [2]. A clinical trial to assess safety and tolerability will be reported soon (NCT04182763). Both interventions are being assessed primarily for their effect on the neurological problems caused by damage to the basal ganglia. But as disease-modifying therapies are developed for PKAN, interventions to preserve vision and combat the associated retinopathy will become increasingly important.

Here we report the longitudinal characterization of the retinopathy in this model, which includes reduced visual performance and reduced photoreceptor thickness compared to wild-type mice. Additionally, retinal perturbations were observed in CoA metabolism and dopamine metabolism pathways, similar to those observed in brain. With a measurable behavioral, structural and mechanistic retinal phenotype, the *Pank2*^-/-^ mouse is an ideal pre-clinical model that can be used to evaluate PKAN therapeutics, especially to treat the retina.

## Materials and methods

### Mice

All animal procedures were performed in accordance with the Association for Research in Vision and Ophthalmology (ARVO) Statement for the Use of Animals in Ophthalmic and Vision Research and approved by the Oregon Health & Science University Institutional Animal Care and Use Committee (OHSU IACUC, TR02_IP00000610). The *Pank2*^-/-^ mice were originally generated by the Gitschier Lab at UCSF [9]. Wild-type littermates were used as controls. All mice were bred in-house and were housed in a specific-pathogen-free animal facility at ambient temperature (22 ± 2 °C), with air humidity 40–70%, and under 12-h light/12-h dark cycles with free access to standard rodent chow diet (5L0D - PicoLab) and water at the Casey Eye Institute, OHSU. All mice used in experiments ranged from 3 to 12 months in age. Both male and female mice were used, pooled and tested collectively throughout the study. The study did not involve separate analyses for each sex. Mice were euthanized by carbon dioxide inhalation at a flow rate of 2–5 liters/minute with secondary euthanasia of cervical dislocation. No masking or randomization was used in the study.

### Spectral domain optical coherence tomography

Mice were sedated by inhalation of 1.5% isoflurane delivered via nose cone, and pupils were dilated with topical 1% atropine sulfate (AKORN, Lake Forest, IL, USA), 2.5% phenylephrine (AKORN, Lake Forest, IL, USA) and 0.5% tropicamide (AKORN, Lake Forest, IL, USA). Mice were seated in a Bioptigen AIM-RAS holder and spectral domain optical coherence tomography (SD-OCT) images were obtained using an Envisu R2200-HR SD-OCT instrument (Bioptigen, Durham, NC, USA) following the application of Systane Lubricant eye drops (9004494−0109, Alcon, Fort Worth, TX, USA) to maintain corneal clarity and hydration [10,11]. Each eye was imaged using temporal and nasal quadrant linear horizontal scans and superior and inferior quadrant linear vertical scans. Nasal and temporal SD-OCT images were manually segmented to calculate outer retinal thickness (REC + , photoreceptors) and total retinal (TR) thickness. Measurements were taken using a caliper 0.5 mm nasally and 0.5 mm temporally from the optic nerve at 5 different locations (0.5, 0.25, 0, −0.25 and −0.5 mm vertically from the optic nerve) [12].

### Histological analysis

After mice were euthanized, a burn mark was made at the superior cornea of the eyes to mark orientation. Eyes were then enucleated, fixed in 4% paraformaldehyde overnight at 4°C, dehydrated in 70% ethyl alcohol, and processed by paraffin embedding. 4 μm-thick sections crossing the optic nerve were cut using a microtome. After deparaffinization in xylene and rehydration in ethyl alcohol, sections were stained with hematoxylin and eosin (H&E) (Fisher Scientific, Hercules, CA, USA) and imaged with a Leica DMI3000 B manual inverted microscope. (Leica Corp., Wetzlar, Germany). Zen Blue lite software (Carl Zeiss Microscopy LLC., White Plains, NY, USA) was used to capture images. All images were taken at 40x magnification.

### Retinal fluorescein angiography

Mice were anesthetized with a mixture of ketamine (100 mg/kg, Mylan Institutional LLC, Rockford, IL, USA) and xylazine (10 mg/kg, MWI, Boise, ID, USA), and pupils were dilated with topical 1% atropine sulfate (AKORN, Lake Forest, IL, USA), 2.5% phenylephrine (AKORN, Lake Forest, IL, USA) and 0.5% tropicamide (AKORN, Lake Forest, IL, USA). Mice were subcutaneously injected with 1 µl/g of 10% fluorescein. Single-image full fluorescein angiograms were obtained six minutes after fluorescein injection. A Micron IV scanning laser ophthalmoscope (Phoenix-Micron Inc., Bend, OR, USA) at a consistent intensity was used to perform FA with mice and seated in a Bioptigen AIM-RAS holder.

### Quantitative RT-PCR

Nine-months old mice were euthanized and dissected retinas were snap frozen using liquid nitrogen. RNeasy Plus Mini Kit (Qiagen) was used to isolate total RNA without genomic DNA contamination following manufacturer’s protocol. After measuring RNA concentration, 0.7ug of total RNA was subject to reverse transcription reaction using SuperScript III First-Strand Synthesis System. Final cDNA samples were diluted in 1/5 with UltraPure water and real-time PCR analyses were performed using PowerUp SYBR Green Master Mix on QuantStudio 3 Real-Time PCR System (all ThermoFisher). All data were obtained and analyzed using Design & Analysis software (ThermoFisher). All target gene data were initially normalized to a housekeeping gene expression (mouse *Gapdh*) and then compared to the wild-type group (Comparative CT Method, ΔΔCT). Primers for this experiment were previously published and listed below in Table 1 [2].

**Table 1:** *Coasy, Tfrc, Drd1* and *Drd2* primer sequences.

Species	Target	Forward	Reverse
Mouse	*Coasy*	GGAGGCCTTTGGAACAGATATT	GAGGATCTTCATCTGCTTCTTGT
Mouse	*Tfrc*	GAGTATCACTTCCTGTCGCCCTATG	GCTGAGAGAGTGTGAGAGCCAGAGC
Mouse	*Drd1*	TCCCAGATCGGGCATTTG	CTCTTCCTGGTCAATCTCAGTC
Mouse	*Drd2*	GCCATTGTTCTTGGTGTGTTC	TAGAGGACTGGTGGGATGTT
Mouse	*Gapdh*	TGCACCACCAACTGCTTAGC	GGCATGGACTGTGGTCATGAG

qPCR primers are listed for each target gene.

### Optokinetic tracking

Visual performance was assessed by measuring optokinetic tracking (OKT) responses using the OptoMotry© system [13,14]. After system calibration, animals were placed on the testing pedestal and allowed to acclimate for 2 minutes before data collection. A simple staircase method was used at 100% contrast to measure visual performance thresholds [13,14]. OKT responses of *Pank2*^-/-^ mice and their WT littermates were measured at 3-, 6-, 9- and 12-month age timepoints.

### Statistical analysis

For gene expression data, two-tailed unpaired Welch’s T tests was performed, comparing gene expression in the neural retinas of *Pank2*^-/-^ mice to those of WT mice normalized to 100% at a confidence interval of 95% and ɑ = 0.05. For retinal thickness and visual performance, ordinary one-way ANOVA with Tukey’s multiple comparisons tests were performed at ɑ = 0.05. Statistical tests were performed using Prism version 10.1.0 (264) for macOS Ventura 13.6 (GraphPad Software, San Diego, CA, USA).

## Results

### *Pank2*^-/-^ mouse retinas have defects in the CoA pathway and dopamine metabolism

**Fig 1:**
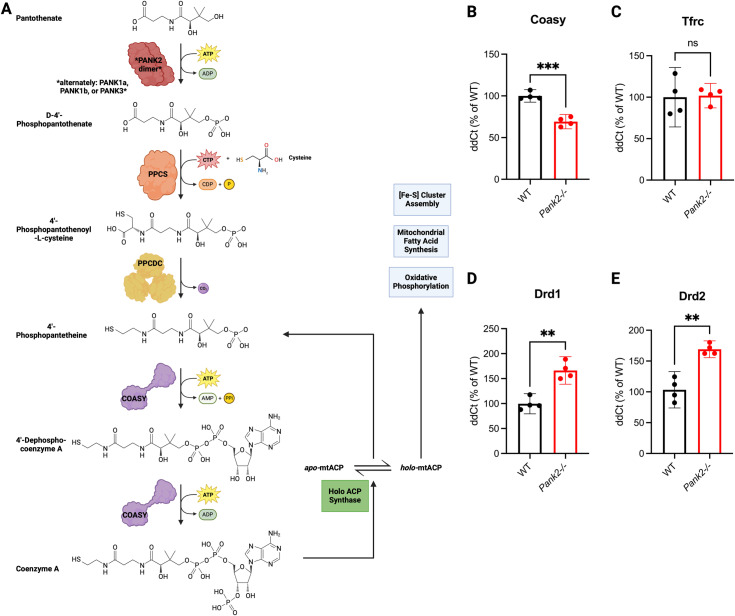
*Pank2*^-/-^ causes differential retinal gene expression in CoA and dopaminergic pathways compared to WT. **(A)** A flow chart depicting the proposed metabolic role of PANK2 in the CoA synthesis pathway. Bar graphs show mean relative quantification of **(B)** *Coasy* (*p* = 0.0002), **(C)** *Tfrc* (*p* = 0.8833), **(D)** *Drd1* (*p* = 0.0011), and **(E)** *Drd2* (*p* = 0.0025) mRNA with 95% confidence interval from the neural retinas of WT (black, n = 4) and *Pank2*^**-/-**^ (red, n = 4) neural retinas collected at 9 months and quantified for mRNA expression relative to WT (100%) by qRT-PCR. Data shown were evaluated by two-tailed unpaired Welch’s t-test. Asterisks represent statistical analyses compared to the WT neural retinas. *P < 0.05, **P < 0.01, ***P < 0.001. Minimal data for qRT-PCR provided in S1 Table.

Among its actions, Pank2 catalyzes phosphorylation of pantothenate to D-4’-phosphopantothenate in the CoA synthesis pathway. (Fig 1A) To investigate potential impacts of *Pank2* knockout on the CoA pathway, iron homeostasis, and dopaminergic pathway in retina, we measured mRNA expression of *Coasy, Tfrc, Drd1,* and *Drd2,* in retinal tissue of *Pank2*^-/-^ mice and WT littermates at 9 months. Like in globus pallidus tissue, retinal *Coasy* transcriptional expression was significantly down-regulated in mutant compared to WT animals, with a fold-change of –0.31, most likely in response to decreased substrate for *Coasy*, encoding CoA synthase, resulting from the defect in pantothenate kinase 2. (Fig 1B, S1 Table) We found no difference in retinal *Tfrc* expression between *Pank2*^-/-^ and WT animals. (Fig 1C, S1 Table) Mirroring *Drd1* and *Drd2* expression in globus pallidus tissue, retinal transcriptional expression of *Drd1* and *Drd2* was significantly up-regulated, with fold-changes of 0.67 and 0.64, respectively, most likely reflecting a lack of ligand and perturbation of retinal dopamine metabolism (Fig 1D and 1E, S1 Table).

### *Pank2*^-/-^ mice have progressively impaired visual performance

**Fig 2:**
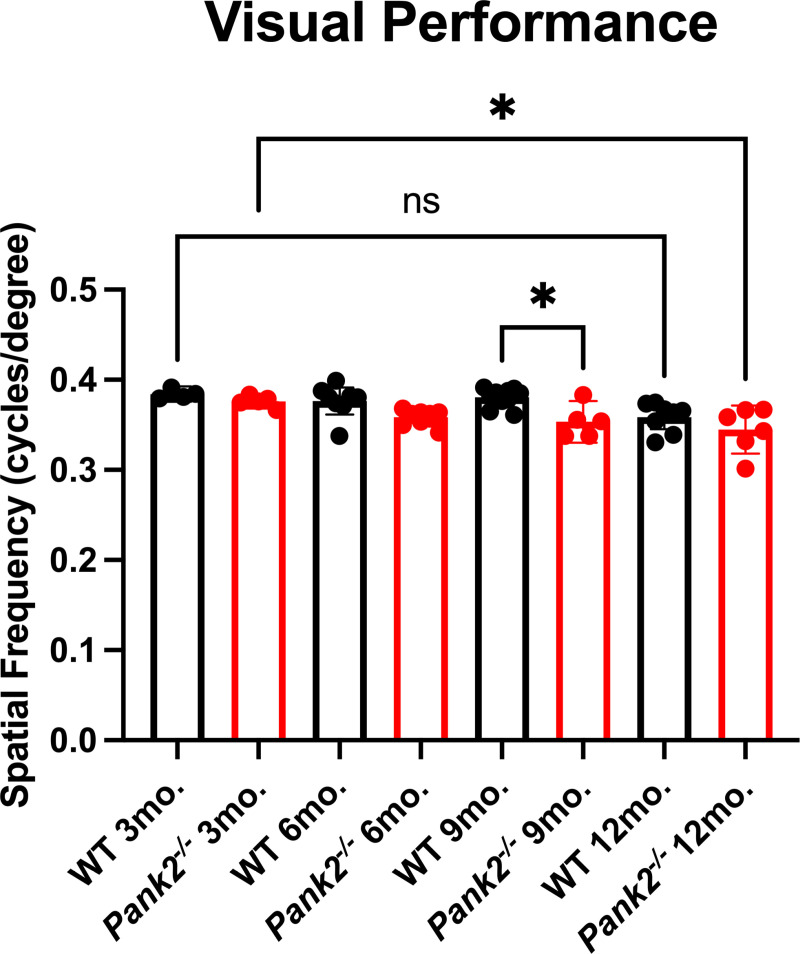
*Pank2*^-/-^ mice have progressive visual performance impairment between 3 and 12 months compared to WT. Bar graphs show mean with 95% confidence interval of optokinetic tracking indicating *Pank2*^*-/-*^ mice (red, n = 5-10) have degeneration in visual performance between 3 and 12 months (*p* = 0.0169) and lower visual performance than WT (black, n = 4-12) at 9 months (*p* = 0.0195). Data shown were evaluated by ordinary one-way ANOVA. Asterisks represent statistical analyses compared to the WT mouse group. *P < 0.05. Statistical data listed in Table 2. Minimal OKT data provided in S2 Table.

To determine if these mice have a visual behavioral phenotype, optokinetic tracking (OKT) was used to measure visual performance progression 3, 6, 9 and 12 months. Visual performance decreased in *Pank2*^-/-^ mice from an average spatial frequency of 0.376 cycles/degree at 3 months to an average spatial frequency of 0.345 cycles/degree at 12 months, suggesting progressive degeneration of retinal function. Additionally, OKT data show lower visual performance of mutant animals at 9 months compared to WT controls, with WT animals averaging a spatial frequency of 0.381 cycles/degree, while *Pank2*^-/-^ animals demonstrated an average a spatial frequency of 0.354 cycles/degree. (Fig 2, Table 2, S2 Table)

**Table 2:** Data table for visual performance comparisons.

Explanatory variable	*P-*value	Test statistic (q-statistic)	Degrees of freedom
WT 3mo. vs. WT 12mo.	0.0983	4.075	50
*Pank2*^*-/-*^ 3mo. vs. *Pank2*^*-/-*^ 12mo.	0.0169	5.047	50
WT 9mo. vs. *Pank2*^*-/-*^ 9mo.	0.0195	4.975	50

### *Pank2*^*-/-*^ mouse retinas have a moderate retinal degeneration

To determine how the retina is affected by these changes in gene expression and to thoroughly characterize any disease progression, we longitudinally assessed the retina through spectral domain – optical coherence tomography (SD-OCT), histology, and fluorescein angiography (FA).

**Fig 3:**
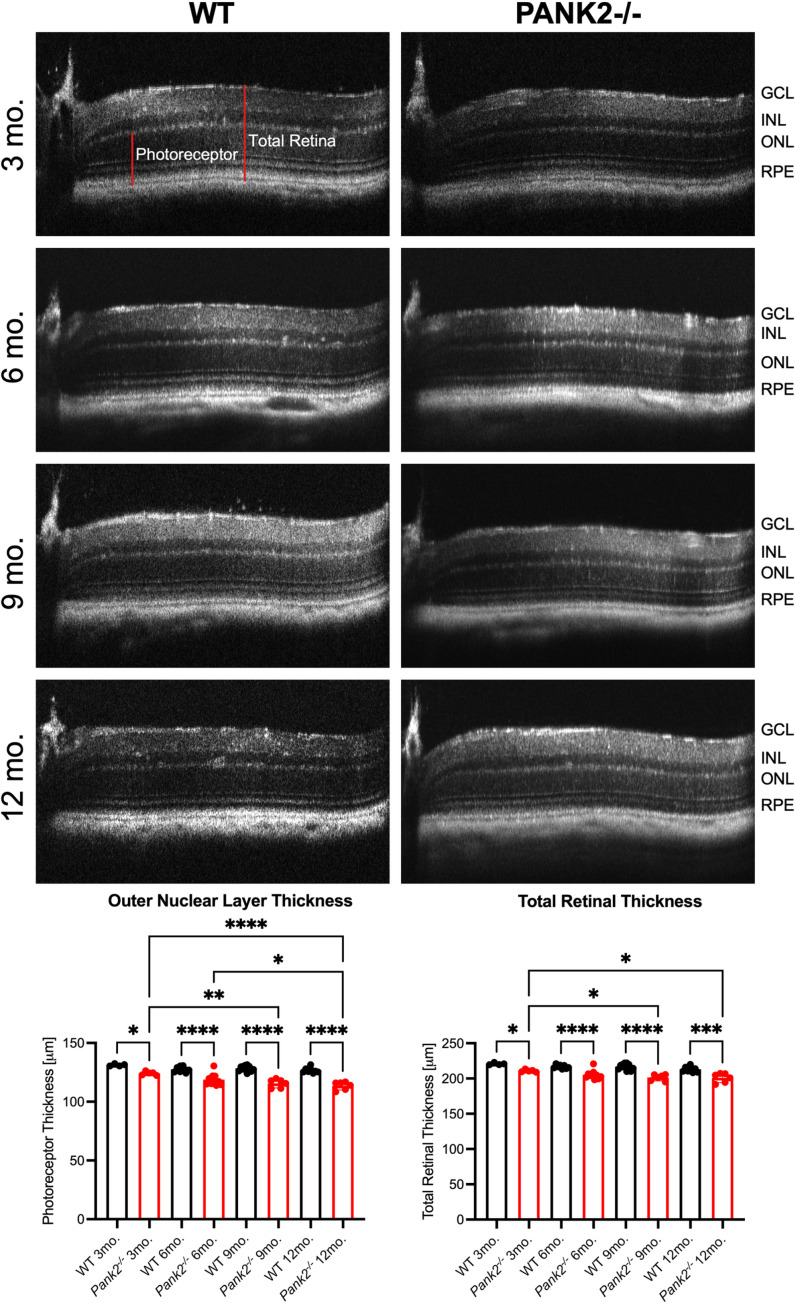
*Pank2*^*-/-*^ mice have progressive retinal thinning compared to WT. Representative SD-OCT images of *Pank2*^*-/-*^ and WT mouse retinas at 3, 6, 9, and 12 months of age. Bar graphs show mean of thickness of ONL and total retina with 95% confidence interval in WT (black, n = 4-12) and *Pank2*^*-/-*^ (red, n = 5-10) mice at 3, 6, 9 and 12 months of age. Data shown were evaluated by ordinary one-way ANOVA. ONL thickness R^2^ = 0.7084. TR thickness R^2^ = 0.7930. *P < 0.05, **P < 0.01, ***P < 0.001, ****P < 0.0001. ONL thickness statistical data listed in Table 3. Total retina thickness statistical data listed in Table 4. Minimal data for ONL and total retina thickness measurements listed in S3 Table.

**Fig 4:**
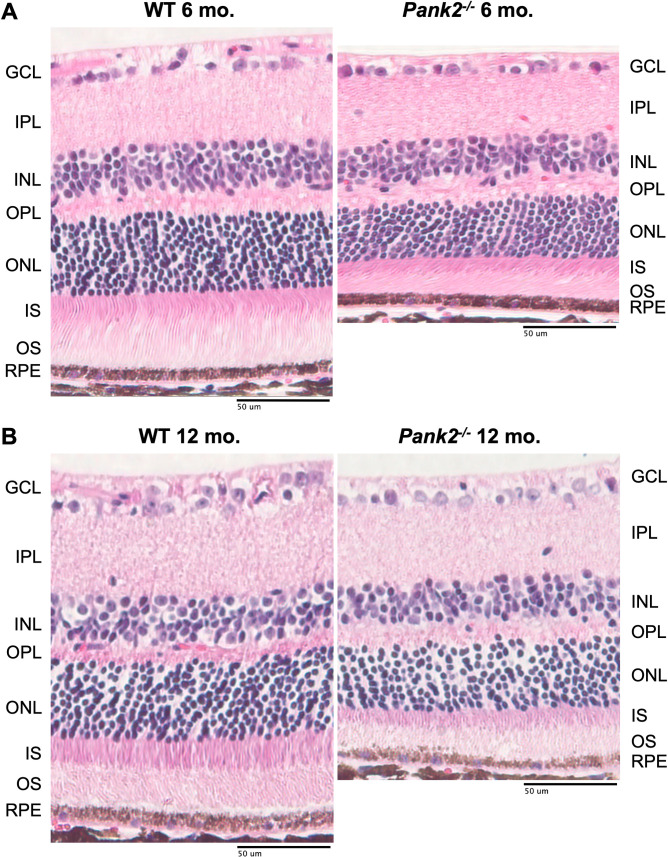
Retinal histology shows thinned outer nuclear layer in *Pank2*^*-/-*^ mice compared to WT. H&E histology data shows significant retinal thinning and photoreceptor loss in *Pank2*^*-/-*^ mice compared to WT mice at **(a)** 6 months and **b)** 12 months (n = 4 for each genotype at each timepoint).

SD-OCT data showed that *Pank2*^*-/-*^ mice had reduced photoreceptor and total retinal thickness compared to their age-matched litter-mate controls from 3 to 12 months (Fig 3, p < 0.05, Tables 3 and 4, S3 Table). Outer nuclear layer (ONL) and total retina thickness was reduced by an average of 10 μm from 3 to 12 months, indicating that photoreceptor loss was responsible for the measured thinning (Fig 3, p < 0.05, Tables 3 and 4, S3 Table). Histological examination of *Pank2*^*-/-*^ and WT mouse retinas at 6 and 12 months shows photoreceptor cell loss and shortened outer segments, leading to overall retinal thinning in *Pank2*^*-/-*^ mouse retinas (Fig 4). Both SD-OCT and histology indicate moderate progressive photoreceptor cell loss.

**Table 3:** Data table for ONL thickness comparisons.

Explanatory variable	*P-*value	Test statistic (q-statistic)	Degrees of freedom
WT 3mo. vs. *Pank2*^*-/-*^ 3mo.	0.0291	4.762	51
*Pank2*^*-/-*^ 3mo. vs. *Pank2*^*-/-*^ 9mo.	0.0010	6.335	51
*Pank2*^*-/-*^ 3mo. vs. *Pank2*^*-/-*^ 12mo.	<0.0001	7.828	51
WT 6mo. vs. *Pank2*^*-/-*^ 6mo.	<0.0001	8.317	51
*Pank2*^*-/-*^ 6mo. vs. *Pank2*^*-/-*^ 12mo.	0.0429	4.553	51
WT 9mo. vs. *Pank2*^*-/-*^ 9mo.	<0.0001	11.54	51
WT 12mo. vs. *Pank2*^*-/-*^ 12mo.	<0.0001	10.93	51

**Table 4:** Data table for total retina thickness comparisons.

Explanatory variable	*P-*value	Test statistic (q-statistic)	Degrees of freedom
WT 3mo. vs. *Pank2*^*-/-*^ 3mo.	0.0379	4.622	51
*Pank2*^*-/-*^ 3mo. vs. *Pank2*^*-/-*^ 9mo.	0.0239	4.865	51
*Pank2*^*-/-*^ 3mo. vs. *Pank2*^*-/-*^ 12mo.	0.0171	5.037	51
WT 6mo. vs. *Pank2*^*-/-*^ 6mo.	<0.0001	7.385	51
WT 9mo. vs. *Pank2*^*-/-*^ 9mo.	<0.0001	9.672	51
WT 12mo. vs. *Pank2*^*-/-*^ 12mo.	0.0002	6.943	51

**Fig 5:**
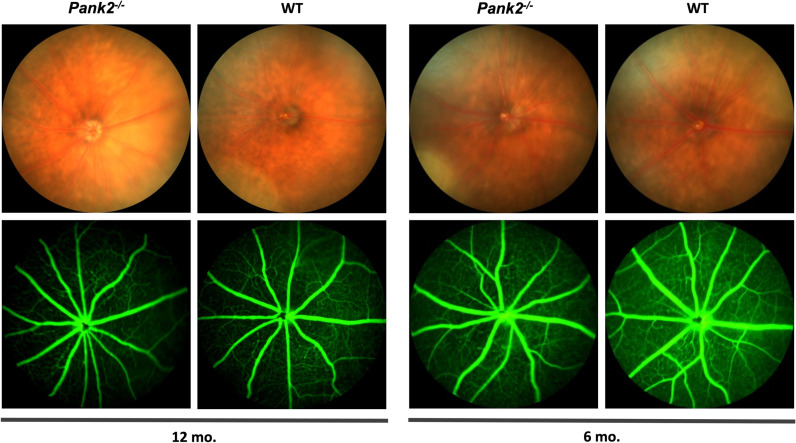
*Pank2*^*-/-*^ mice have no changes to retinal vasculature compared to WT. Fluoresceine Angiography data of 6-month-old *Pank2*^*-/-*^ (n = 10) and WT (n = 8) mice and 12-month-old *Pank2*^*-/-*^ (n = 5) and WT (n = 6) mice shows no significant differences or degeneration in retinal vasculature.

We sought to determine if the degeneration of murine *Pank2*^*-/-*^ photoreceptors may be correlated with changes to retinal vascular. To analyze retinal vasculature, we performed fluorescein angiography on the *Pank2*^*-/-*^ and WT mice at 6 and 12 months. The fluorescein angiograms showed no obvious dropout in retinal vascular structure at either time point, suggesting that retinal vasculature is not affected in *Pank2*^*-/-*^ mice, consistent with the retinal phenotype in people with PKAN (Fig 5).

## Discussion

We have expanded knowledge of the retinopathy phenotype in a mouse model of PKAN. Previous studies of these mice by Kuo *et al*. (2004) identified a diminished pupillary response, and a progressive decline in photoreceptor function by electroretinogram (ERG)[9]. Our work focused on repeatable, longitudinal retinal exams that may serve therapeutics development. We report abnormal transcription of CoA- and dopamine-related biomarkers in the retina and normal expression of *Tfrc* transcripts, encoding the key iron importer. This model recapitulates key features of the human retinopathy, including progressive visual behavior impairment, retinal thinning and photoreceptor degeneration, with apparently normal retinal vasculature. With a measurable behavioral, structural and mechanistic retinal phenotype, the *Pank2*^*-/-*^ mouse model represents a tractable system with which to investigate pathogenesis of the PKAN retinopathy and evaluate candidate therapeutics.

Retinopathic changes in people with PKAN include retinal thinning, observed by SD-OCT, particularly in the ONL, and correlate with a loss of photoreceptors (early onset loss of rods followed by later onset loss of cones) that lead to abnormal electroretinography results with a specific reduction in b-wave scotopic amplitudes and abnormal retinal pigmentary changes visible with funduscopic examination. These clinical assessments can serve to quantify the progressive visual impairment in PKAN [1,8,15]. PKAN-associated pigmentary retinal degeneration typically occurs early in PKAN pathogenesis and tends to follow a typical clinical course of night blindness followed by progressive loss of peripheral vision, constricting the visual field and eventual blindness. The rate of progression of PKAN is correlated to the age of onset but is nonuniform, with periods of stability interspersed with episodes of deterioration of symptoms with no known cause or trigger. The average age of onset for classic PKAN is 3.4 years, and the average age of onset for atypical PKAN is 13.6 years [16].

Murine disease models that mimic human clinical manifestations of the disease are highly valued in research. However, while this model captures some retinal phenotype, its retinal degeneration occurring over the majority of its lifespan being much slower and milder than that of human PKAN is not wholly analogous to the speed and severity at which human PKAN retinopathy progresses. While the progression of visual impairment in this model is slow, SD-OCT data show definitive changes to retinal morphology, particularly with reduction in ONL thickness compared to WT and degeneration of ONL and total retinal thickness between 6-month timepoints. The reduced ONL thickness models human disease, with a recent study showing human PKAN patients with thinned retinas, especially in the ONL region [8]. Our detailed and longitudinal characterization of the progressive retinopathy in *Pank2*^*-/-*^ mice establish *Pank2*^*-/-*^ mice as a model for PKAN retinal pathogenesis.

Current treatments for PKAN are limited in effect although new approaches are under investigation. Current clinical treatments include deep brain stimulation surgery, intrathecal baclofen pumps, and iron chelation medications [17]. However, none of these treat or correct the core issue of impaired CoA metabolism in PKAN. Thus, much pre-clinical research has focused on targeted replenishment of CoA, such as by treatment with pantetheine or the pantothenate kinase activator, BBP-671 [4,18,19]. The most promising potential treatment of bypassing the impaired CoA metabolism in PKAN is dietary supplementation of 4′‐phosphopantetheine, a CoA pathway intermediate. In *Pank2*^*-/-*^ mice, dietary supplementation of 4′‐phosphopantetheine was able to correct the CoA, iron and dopamine metabolic defects and molecular perturbations present in untreated *Pank2*^*-/-*^ mice [2]. Dietary supplementation of 4′‐phosphopantetheine in PKAN patients is being investigated in a phase 2 clinical trial at Oregon Health & Science University (NCT04182763).

As life-preserving therapies for PKAN are developed, treatments for PKAN retinopathy will become more important to preserve the vision and quality of life for PKAN patients. Therefore, an animal model of PKAN retinopathy is necessary to test potential retinopathy therapeutics. Our lab is interested in generating and evaluating local retinal gene therapies, including *Pank2* gene augmentation and base editing, using this *Pank2*^*-/-*^ mouse model. Additionally, studies are needed to determine if the systemic therapeutic effects conferred by 4′‐phosphopantetheine supplementation extend to the retina or are limited to extraocular tissues. This *Pank2*^*-/-*^ mouse model recapitulates some aspects of human PKAN retinopathy and can therefore serve as a useful model for assessing PKAN retinopathy therapeutics.

## Supporting information

S1 Table Extended qRT-PCR data for Fig 1B-1D. (XLSX)

S2 Table Extended optokinetic tracking data for Fig 2. (XLSX)

S3 Table Extended ONL and total retina thickness data for Fig 3. (XLSX)
